# Esketamine Prevents Postoperative Emotional and Cognitive Dysfunction by Suppressing Microglial M1 Polarization and Regulating the BDNF-TrkB Pathway in Ageing Rats with Preoperative Sleep Disturbance

**DOI:** 10.1007/s12035-023-03860-4

**Published:** 2024-01-15

**Authors:** Yuxin Wen, Jiawen Xu, Jiahong Shen, Zili Tang, Shuxin Li, Qun Zhang, Jiaqi Li, Jianliang Sun

**Affiliations:** 1 Zhejiang University School of Medicine, Hangzhou, China; 2 Department of Anesthesiology, Affiliated Hangzhou First People’s Hospital, School of Medicine, Westlake University, Hangzhou, China; 3 Department of Anesthesiology, The Fourth Clinical School of Medicine, Zhejiang Chinese Medical University, Hangzhou, China; 4 School of Second Clinical Medical College, Wenzhou Medical University, Wenzhou, China

**Keywords:** Esketamine, Perioperative sleep deprivation, Postoperative depression, Postoperative cognitive dysfunction, Microglial M1-polarization, BDNF-TrkB signalling

## Abstract

Postoperative depression (POD) and postoperative cognitive dysfunction (POCD) have placed heavy burden on patients’ physical and mental health in recent years. Sleep disturbance before surgery is a common phenomenon that has been increasingly believed to affect patients’ recovery, especially in aged patients, while little attention has been paid to sleep disruption before surgery and the potential mechanism remains ambiguous. Ketamine has been reported to attenuate POCD after cardiac surgery and elicit rapid-acting and sustained antidepressant actions. The present study aimed to clarify the effect of esketamine’s (the S-enantiomer of ketamine) protective effects and possible mechanisms of action in POCD and POD. Our results showed that sleep disturbance before surgery exacerbated microglial M1 polarization and microglial BDNF-TrkB signalling dysfunction induced by surgery, resulting in postoperative emotional changes and cognitive impairments. Notably, treatment with esketamine reversed the behavioural abnormalities through inhibiting the M1 polarization of microglia and the inflammatory response thus improving BDNF-TrkB signalling in vivo and vitro. In addition, esketamine administration also reversed the impaired hippocampal synaptic plasticity which has been perturbed by sleep disturbance and surgery. These findings warrant further investigations into the interplay of esketamine and may provide novel ideas for the implication of preoperative preparations and the prevention of postoperative brain-related complications.

**Supplementary Information:**

The online version contains supplementary material available at 10.1007/s12035-023-03860-4.

## Background

It has been reported that approximately 15% of patients undergoing various surgeries are at higher risk of central nervous system (CNS) complications, leading to 80% of all deaths during the perioperative period [1]. Anaesthesia, anxiety, and pain are highly likely to lead to postoperative abnormal behaviours, including anxiety, depression, chronic pain, and cognitive dysfunction [2]. Increasing evidence supports the idea that many patients will develop postoperative depression-like symptoms (POD) after surgery, including after orthopaedic surgery, cardiac surgery, and bariatric surgery [3–5]. Depression postoperatively is often accompanied by postoperative cognitive impairment (POCD) [1]. However, little academic attention has been focused on neither POD nor POCD, and their pathogenesis and therapeutic mechanisms remain unknown.

Sleep disorders affect many patients, especially the elderly, and are a highly encountered phenomenon affecting millions of depressed patients worldwide [6]. A growing amount of evidence supports the fact that sleep loss modifies the immune response and affects the levels of pro- and anti-inflammatory molecules. Sleep disturbance is commonly observed before surgery. There is increasing recognition of the potential link between sleep disturbances and perioperative neurocognitive disorders, but the mechanisms and signalling pathways involved have scarcely been studied.

Microglia, which act as the primary resident immune cells at the first line of defence in the brain [7], have attracted great attention in recent decades. Within the brain, activated microglia have two main phenotypes: the proinflammatory M1 phenotype and the anti-inflammatory M2 phenotype. M1-polarized microglia are usually induced by proinflammatory stimuli and adopt a proinflammatory state that produces proinflammatory cytokines, such as tumour necrosis factor-α (TNF-α), interleukin-1β (IL-1β), interleukin-6(IL-6), nitric oxide (NO), and reactive oxygen species (ROS), which are consistently upregulated in depression patients [8, 9]. Studies have shown that the occurrence of neuroinflammation and the number and activation status of microglia can lead to spatial learning and memory impairment [10] as well as depression [11]. Numerous studies have shown that inhibiting microglial overactivation, reducing proinflammatory cytokines released by microglia and increasing microglia-associated neurotrophic factors play key roles in neurogenesis and cognitive functions [12, 13]. Brain-derived neurotrophic factor (BDNF) is the most important neurotrophic factor released by nerve cells and microglia and performs as a chief regulator of axonal growth, neuronal differentiation, survival, and synaptic plasticity [14]. It has been found that microglia-dependent BDNF plays a key role in learning and memory impairment [13, 15].

Esketamine, an enantiomer of (R, S)-ketamine (ketamine), has a stronger affinity for NMDA receptors and a higher anaesthetic potency than ketamine [16]. Acting on neurotransmitter systems, esketamine also has significant analgesic effects and is widely used as an effective sedative analgesic drug clinically [17]. Regarding the effects of esketamine, increasing evidence demonstrates that the rapid and long-lasting antidepressant effects require the inhibition of microglial activation in the hippocampus [18] and rapid transient translation of BDNF [19]. However, as a sedative, an analgesic, and an antidepressant, whether esketamine could benefit postoperative cerebral complications after sleep deprivation has not been investigated.

In the present study, we used aged rat models with preoperative sleep disturbance to address whether esketamine could improve postoperative cerebral complications both cognitively and emotionally by inhibiting microglial overactivation and promoting BDNF secretion. It should be noted that, unlike previous approaches, we employed a model of intermittent sleep deprivation (12 h sleep deprivation lasting for 3 days). Because rats are generally preponderantly awake during the dark phase and asleep during the light phase [20], these modifications were made to more closely approximate to the experience of sleep loss in human subjects [21].

## Materials and Methods

### Animal and Drug Treatment

Aged (24-month-old) male Sprague–Dawley rats weighing 600–700 g were used in the study and obtained from Zhejiang Chinese Medical University Laboratory Animal Research Center. Rats were kept in groups of five per cage and maintained under standard environmental conditions of temperature (25 ± 2 °C) and humidity (55 ± 2%). All rats had free access to food and water. All experiments followed the guidance of the Animal Ethics Committee of Zhejiang Chinese Medical University. Esketamine was purchased from Jiangsu Hengrui Medicine Co., Ltd. (50 mg/2 mL, Jiangsu, China), and was dissolved in normal saline.

### Sleep Disturbance and Surgery

Sleep disturbance was initiated at lights on (8 AM) until lights off (8 PM) and lasted for 3 days. The deprivation was performed through gentle handling, directly observing the rat’s motor activity and gently stroking the fur with a brush when no activity was observed [22]. The surgery was performed 1 day after sleep deprivation. An aseptic exploratory laparotomy was performed under sevoflurane anaesthesia (7–8% for induction and 3–4% for maintenance). The abdominal region was shaved and sterilized with iodophor and ethanol. The abdominal cavity was fully exposed by a vertical incision of 3 cm at approximately 1 cm below the ensisternum. The surgeon successively explored the liver, spleen, kidney, stomach, and intestine for 5 min respectively. Approximately 10 cm of the intestine was then exteriorized and rubbed between the thumb and index finger for 30 s. The intestine was then returned to the peritoneal cavity before the incision was sutured with sterile 4-0 sutures and disinfected with iodophor, and the wound was dressed with sterile gauze. The total duration of the operation was approximately 30 min. The rats in the control group and esketamine group were anaesthetized, shaved and disinfected as described above, but no incision was made. The duration of anaesthesia was the same as that in the surgical groups.

### Experimental Design

**Fig. 1:**
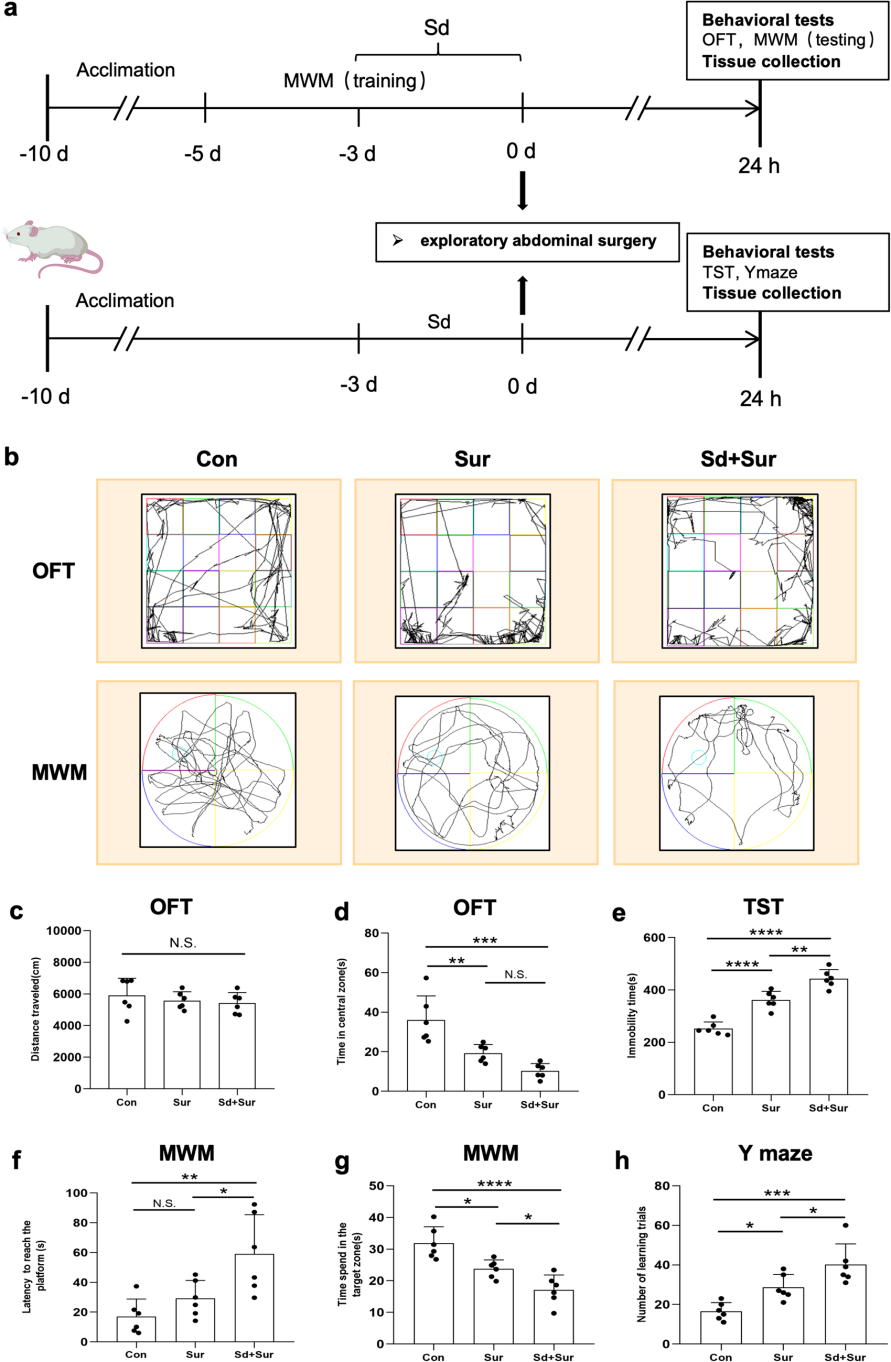
Sleep disturbance enhanced postoperative cognitive impairment and postoperative depression-like symptoms. **a** Schematic timeline of experiment 1 procedure. **b** The track plot of the OFT and MWM performances of rats. OFT: **c** the overall travel distance and **d** time spent in the central zones. TST: **e** the immobility time. MWM: **f** latency; **g** time spent in the target zone. Y maze: **h** the number of learning trials. Data are shown as mean ± S.E.M of six rats in each group. N.S: *p* > 0.05; *p < 0.05; **p < 0.01; ****p < 0.0001

This research was conducted in three separate experiments (shown in Figs. 1a and 3a).

#### Experiment 1

Rats were allocated randomly to three groups: (a) Con group: anaesthesia; (b) Sur group: anaesthesia + surgery; (c) Sd+Sur group: sleep disturbance + anaesthesia + surgery. Behavioural tests and brain tissue collection were performed 24 h after the operation. The study design is briefly illustrated in Fig. 1a.

#### Experiment 2

Rats were randomly assigned to four groups: (a) Con group: anaesthesia + i.p. normal saline; (b) Sd + Sur group: sleep disturbance + anaesthesia + surgery + i.p. normal saline; (c) Sd + Sur + Sk group: sleep disturbance + anaesthesia + surgery + i.p. esketamine; (d) Sk group: anaesthesia + i.p. esketamine. The esketamine group was injected with esketamine 30 min after the surgery, and the saline group was injected with an equal volume of 0.9% saline. Behavioural tests and brain tissue collection were performed 24 h after the operation. Referring to previous experiments, 10 mg/kg esketamine was selected to explore the effects on our rat model [23–25]. The study design is briefly illustrated in Fig. 3a.

### Cell Cultivation and Cell Viability

Rat primary microglia culture was prepared according to a previously described protocol with slight modifications [26]. Briefly, whole brains were isolated from postnatal (P1–P2) Sprague–Dawley rats. The meninges and blood vessels were completely removed in a cold phosphate-buffered saline (PBS). Then, the brains were minced with sterile scissors and digested with 0.25% trypsin-EDTA solution for 10 min at 37 °C. The trypsinization was stopped by adding the high-glucose DMEM containing 10% FBS. Then, the cells were sieved through a 100-μm sterile mesh, centrifuged at 1500 rpm for 5 min, resuspended and seeded in 25 cm^2^ culture flasks, and cultured at 37 °C and 5% CO_2_/95% air for 10 days. Confluent 10-day-old mixed glial cultures were placed on a shaker at 120 rpm for 2.5 h to induce detachment of the microglial cells, and the supernatant containing the microglia was collected. The cells were verified by Iba1 (1:500; catalogue: No. 011-27991; Fujiflim, Tokyo, Japan) immunostaining, confirming that more than 95% of the cells were microglia.

Microglial viability after 24 h of continuous exposure to different concentrations of esketamine (1-500 μM) was measured by Cell Counting Kit-8 (CCK8; catalogue: AR1160; Beyotime, Shanghai, China) according to the manufacturer’s protocols. Viability after treatment with 10 ng/mL LPS (catalogue: *Escherichia coli* O111:B4; Sigma-Aldrich, MO, USA) for 30 min, followed by the addition of esketamine (1–500 μM), was tested by CCK8 after 24 h exposure to choose the most appropriate stimulation concentration. The optical density (OD) of each well was measured at a wavelength of 450 nm using a microplate reader.

### Behavioural Studies

#### Tail Suspension Test (TST)

Rats were subjected to the TST as described previously with slight modifications [27]. In the TST experiment, the rats were suspended in an experimental apparatus with a height of approximately 60 cm. A 3-cm section of the rat’s tail was secured by adhesive tape and hung on the protruding hook. Immobility time was recorded in 10 min. The researcher made a judgement based on keeping the two front paws of the rat as the standard, and all immobility times were recorded by the same person.

#### Y Maze Test (Y Maze)

The Y maze was composed of three arms (regions I–III, 8 × 30 × 15 cm) separated by an angle of 120°. The Y maze was conducted according to the previous report, with slight modifications [28]. Every arm has a lamp at the distal end. The safe region was associated with illumination, while the other regions featured electrical foot stimulation (30 V). After 3 min of adaptation, the test was started. The illuminated arm was randomly chosen, while the other arms started electrical foot stimulation 2 s later. A reaching time within 10 s was considered successful. After each stimulation, the next training was started until the rats reached the illuminated arm and stayed there for 15 s. If 9 responses were correct in 10 consecutive foot stimulations, the rats were considered to have satisfied the learning criterion. The total number of stimuli during the training period was recorded.

#### Open Field Test (OFT)

The OFT apparatus was an opaque cube box 100 cm × 100 cm × 40 cm in size. OFT was conducted as previously described with minor modifications [29]. Each rat was placed in the centre of the box with its back facing the researcher and was allowed to explore freely for 5 min. Then, the alcohol was used to remove the smell from the arena before the next rat started. The video tracking system automatically recorded the trajectory, travelling distance, and duration in the open field.

#### Morris Water Maze (MWM) Test

The water maze consisted of a pool containing opaque water (22 ± 1 °C) and a platform (10 cm in diameter) submerged 1.0 cm under the water. The MWM test was performed as previously reported, with minor modifications [30]. The pool was divided into four quadrants, and the target zone was located in a target quadrant (SW). During the training trials, rats were gently put into the water from different quadrants to swim until they found the hidden platform. The rats were allowed to swim for 2 min to find the platform, and they stayed on it for 15 s upon finding the platform. Rats unable to locate the platform were guided to it and stayed at the platform for 15 s. The rats were trained four times per day over five consecutive days. Twenty-four hours after the surgery, the rats were tested for memory retention in a probe trial in the absence of the platform. The performance of each rat was monitored using a digital video camera mounted over the maze and automatically recorded via a video tracking system.

#### Western Blot (WB)

Hippocampal tissues were grated and homogenized with RIPA buffer (catalogue: P0013B; Beyotime, Shanghai, China) with a protease and phosphatase inhibitor cocktail (catalogue: P1050; Beyotime, Shanghai, China) at 4 °C for 20 min. The lysate was centrifuged and the concentration of the extracted supernatant was measured using a BCA kit (catalogue: P0012; Beyotime, Shanghai, China). The protein samples were denatured by sodium dodecyl sulphate-polyacrylamide gel (SDS–PAGE) sample buffer and then transferred to polyvinylidene fluoride (PVDF) membranes. Subsequently, the membranes were blocked with 5% BSA (catalogue: G5001; Servicebio, Wuhan, China) for 1 h and incubated with the primary antibodies overnight at 4 °C. The following primary antibodies were used: Iba1 (1:500, catalogue: catalogue: No.011-27991; Fujiflim, Tokyo, Japan), p-p65 (1:1000, catalogue: #3033, Cell Signaling Technology (CST), Danvers, MA, USA), p-65 (1:1000, catalogue: #8242, CST, Danvers, MA, USA), BDNF (1:1000, catalogue: BM4113, Boster, Wuhan, China), TrkB (1:1000, catalogue: Mo1388-3, Boster, Wuhan, China), postsynaptic density protein 95 (PSD95) (1:1000, catalogue: BM4274, Boster, Wuhan, China), synaptophysin (SYP) (1:1000, catalogue: BM4152, Boster, Wuhan, China), CD86 (1:500, catalogue: sc-28347, Santa Cruz Biotechnology, CA, USA), and β-actin (1:1000, catalogue: BM0627, Boster, Wuhan, China). After washing with TBST (catalogue: G0004, Servicebio, Wuhan, China), we incubated the blots with anti-mouse horseradish peroxidase (HRP)-conjugated IgG antibody H&L (1:1000, catalogue: BA1050, Boster, Wuhan, China), anti-rabbit HRP-conjugated IgG antibody H&L (1:1000, catalogue: BA1054, Boster, Wuhan, China), or anti-goat HRP-conjugated IgG antibody H&L (1:1000, catalogue: BA1060, Boster, Wuhan, China) for 1 h at room temperature. Finally, these protein bands were visualized by the ECL system. Densitometry quantification of bands was performed with ImageJ software (NIH, Bethesda, MD, USA).

#### Immunohistochemistry (IHC)

For the immunohistochemical assay, the sections were incubated with peroxidase blocking reagent for 10 min at room temperature followed by washing three times with PBS for 3 min. Then, the sections were incubated with BDNF antibody (1:50, catalogue: BM4113, Boster, Wuhan, China) at 4 °C overnight and rinsed with PBS three times. Secondary antibody (1:500, catalogue: G1216-3, Boster, Wuhan, China) incubation was performed for 30 min at room temperature. After washing with PBS three times, the samples were mixed with ready-prepared 3,3′-diaminobenzidine (catalogue: Kit9901, Maixin Biotechnique, Fuzhou, China) and washed with tap water to end the reaction. Dehydrating with gradient alcohol, clearing with xylene, and mounting with neutral gum were continued successively. Finally, the sections were observed and photographed using Nikon.

#### Immunofluorescence (IF)

Brains were harvested at 24 h after the operation and excised and postfixed in 4% paraformaldehyde for 72 h. Then, the brain tissues were serially sectioned using a freezing microtome with a thickness of 5 μm. The sections were fixed and blocked with 3% BSA (catalogue: G5001; Servicebio, Wuhan, China) for 30 min. Primary antibodies anti-Iba1(1:500, catalogue: No.011-27991; Fujiflim, Tokyo, Japan) and anti-BDNF (1:50, catalogue: BM4113, Boster, Wuhan, China) were used for immunofluorescence analysis. After incubation at 4 °C overnight, a fluorescent secondary antibody and DAPI were used. Fluorescence images were acquired using fluorescence microscopy (Nikon Corporation).

Microglial cells were seeded into fluorescent dishes and were fixed with 4% paraformaldehyde and 0.1% Triton X-100 solution. After blocking with 5% BSA, cells were then immunostained for the following primary antibodies: p-p65 (1:1600, catalogue: #3033, CST, Danvers, MA, USA), Iba1 (1:500, catalogue: No.011-27991; Fujiflim, Tokyo, Japan), CD86 (1:100, catalogue: sc-28347, Santa Cruz Biotechnology, CA, USA), and BDNF (1:50, catalogue: BM4113, Boster, Wuhan, China). Then, the following experimental procedure is described above and is the same as the animal fluorescence procedure.

#### Enzyme-Linked Immunosorbent Assay (ELISA)

The levels of TNF-α and IL-6 in serum and hippocampal sections of rats were measured. Blood samples were drawn from the left apical rupture of the rat’s heart. The hippocampal samples were sonicated in RIPA buffer containing protease inhibitors. After centrifugation at 4 °C and 12,000 rpm for 20 min, the supernatants were harvested for ELISA. Cytokine concentrations were quantified using enzyme-linked immunosorbent assay kits (catalogue: BMS622, KMC0061, Invitrogen, CA, USA) according to the manufacturer’s protocol.

#### Golgi-Cox Staining

The brains were harvested 24 h after surgery for Golgi staining using a Golgi-cox staining kit (catalogue: G1069, Servicebio, Wuhan, China). Selecting the hippocampal sections, the brain tissues were cut into 2-mm-thick tissue blocks and completely submerged in Golgi-cox staining solution (catalogue: G1069-1, Servicebio, Wuhan, China) for 14 days. Next, the tissues were cut into 100-micron sections with an oscillating microtome (Thermo Fisher, USA) and pasted on gelatine slides. After being concentrated, washed, and sealed, the tissue slides were inspected by an optical microscope (Nikon, Japan).

### Statistical Analysis

All results are presented as the mean ± SEM. The normality of data was evaluated individually. Brown-Forsythe test was used to verify the homogeneity of variance. Differences between groups of normally distributed data were determined by one-way analysis of variance (ANOVA) followed by Tukey’s multiple comparisons test. If the variance was unequal, Brown-Forsythe and Welch ANOVA followed by Games-Howell’s multiple comparison test were used. Differences between groups of non-normally distributed data were tested by Kruskal-Wallis test with multiple comparisons. All statistical charts were drawn using GraphPad Prism statistical software 8.0. *P* < 0.05 was considered statistically significant.

## Results

### Sleep Disturbance Enhanced Postoperative Cognitive Impairment and Postoperative Depression-Like Symptoms of Aged Rats

We evaluated the effect of preoperative sleep disturbance in an aged rat model of laparotomy under anaesthesia. To reduce the interference between different behavioural tests, we conducted them two by two (Fig. 1a). The track plots for the OFT and MWM are presented in Fig. 1b. The OFT and TST are classical tests used to evaluate depression-like behaviours and antidepressant effects [31]. There was no difference in travelled distance between the three groups, indicating that motor ability was not affected among the different groups (Fig. 1c). Compared with controls, rats in the surgery group and sleep disturbance before surgery group spent less time in the central zones (Fig. 1d). Rats in the Sd + Sur group spent the shortest time in the centre. In the TST, the immobility time was significantly increased in the surgery group, and the rats suffering from preoperative sleep disturbance exhibited a longer immobility time (Fig. 1e). The MWM and Y maze have been used to evaluate spatial memory and learning ability in rats [32]. Rats that receiving sleep disturbance exhibited significantly higher escape latency during the learning phase during days 4–5 (Fig. S1a). In the testing period, all groups showed no significant difference in the average swimming speed (Fig. S1b). In this study, there was a notable increase in the latency (Fig. 1f) and a marked decrease in the time spent in the island zone (Fig. 1g) in the surgery and sleep disturbance before surgery group. Furthermore, in the sleep disturbance before surgery group, rats showed a more notable increase in learning trials compared with those in the surgery group and control group (Fig. 1h). Above all, the results support that aged rats suffering from surgery and anaesthesia exhibit postoperative cognitive impairments and depression-like behaviours, and preoperative sleep disturbance could aggravate these behaviours.

### Preoperative Sleep Disturbance Augmented Microglial Activation and Attenuated BDNF-TrkB Signalling in the Hippocampus of Aged Rats

**Fig. 2:**
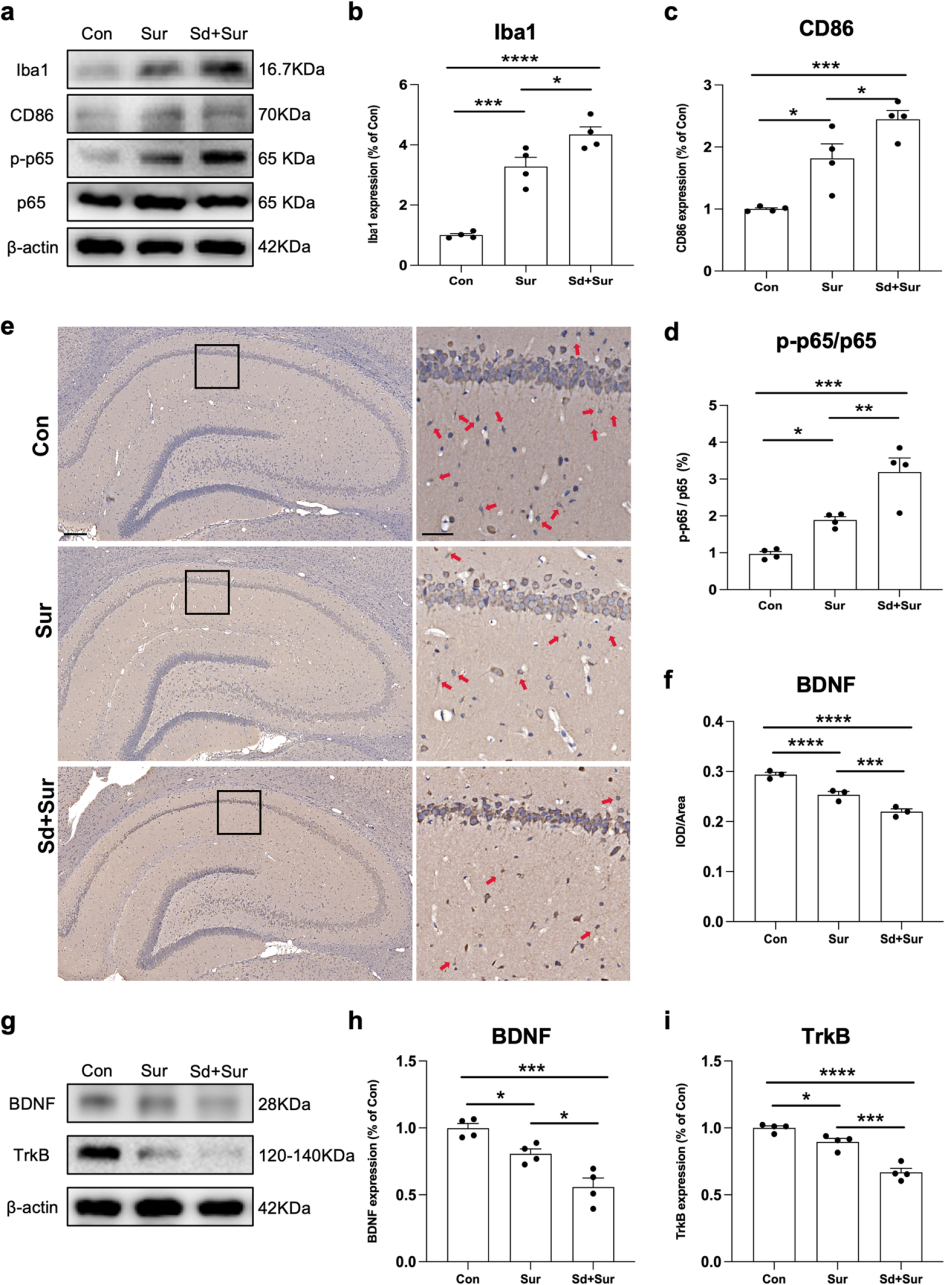
Sleep disturbance enhanced microglial activation and M1-polarization, decreasing the BDNF-TrkB signalling in the hippocampus. **a** Representative results were obtained from an experiment of Iba1, CD86, p-p65 and p65. **b**–**d** Quantification of Iba1, CD86, and p-p65/p65 was expressed as the ratio (in percentage) of the control group. β-actin was used as a loading control (*n* = 4 per group). **e** The expression of BDNF in the hippocampus in Con, Sur, and Sd + Sur groups by IHC. Scale bars 200 μm and 50 μm. **f** IHC analysis of BDNF expression in right and left hippocampus of Con, Sur, and Sd + Sur groups (*n* = 3 per group). **g** Representative results obtained from an experiment of BDNF and TrkB. **h**, **i** Quantification of BDNF and TrkB were expressed as the ratio (in percentage) of the control group. β-actin was used as a loading control (*n* = 4 per group). All data were expressed as means ± SEM. *p < 0.05; **p < 0.01; ***p < 0.001; ****p < 0.0001

Microglia play an important role in the development of neuroinflammation in several central nervous system diseases. To examine the effect of preoperative sleep disturbance on microglial activation, we detected the microglia-specific marker Iba-1 and the M1-polarized microglial marker CD86 in the hippocampus. Our results showed that preoperative sleep disturbance led to a notable increase in microglial activation compared with surgery alone (Fig. 2b). Moreover, the expression of CD86 in microglia was upregulated after exposure to sleep disturbance before surgery (Fig. 2c). NF-κB is a well-recognized regulator of the inflammatory response signalling pathway that is closely related to neuroinflammation and increases the expression of proinflammatory cytokines [33]. The expression of p-p65 and p65 was measured by Western blotting (Fig. 2d). The results showed that exposure to preoperative sleep deprivation promoted the phosphorylation of p65 and increased the p-p65/p65 immunocontent. To determine the role of BDNF-TrkB signalling in POD and POCD, we detected the level of BDNF by immunohistochemistry staining. The results revealed that the protein level of BDNF in the sleep disturbance group was significantly lower than that in the surgery alone and control groups (Fig. 2 e and f). In addition, we detected the protein expressions of BDNF and TrkB in the hippocampus by Western blotting. Sleep disturbance exposure significantly decreased the levels of BDNF and TrkB (Fig. 2g–i). These results indicated that preoperative sleep deprivation aggravated microglial activation and neuroinflammation induced by surgery and attenuated BDNF-TrkB signalling in the hippocampus.

### Esketamine Relieved Postoperative Cognitive Impairment and Attenuated Postoperative Depression-Like Symptoms of Aged Rats with Sleep Disturbance

**Fig. 3:**
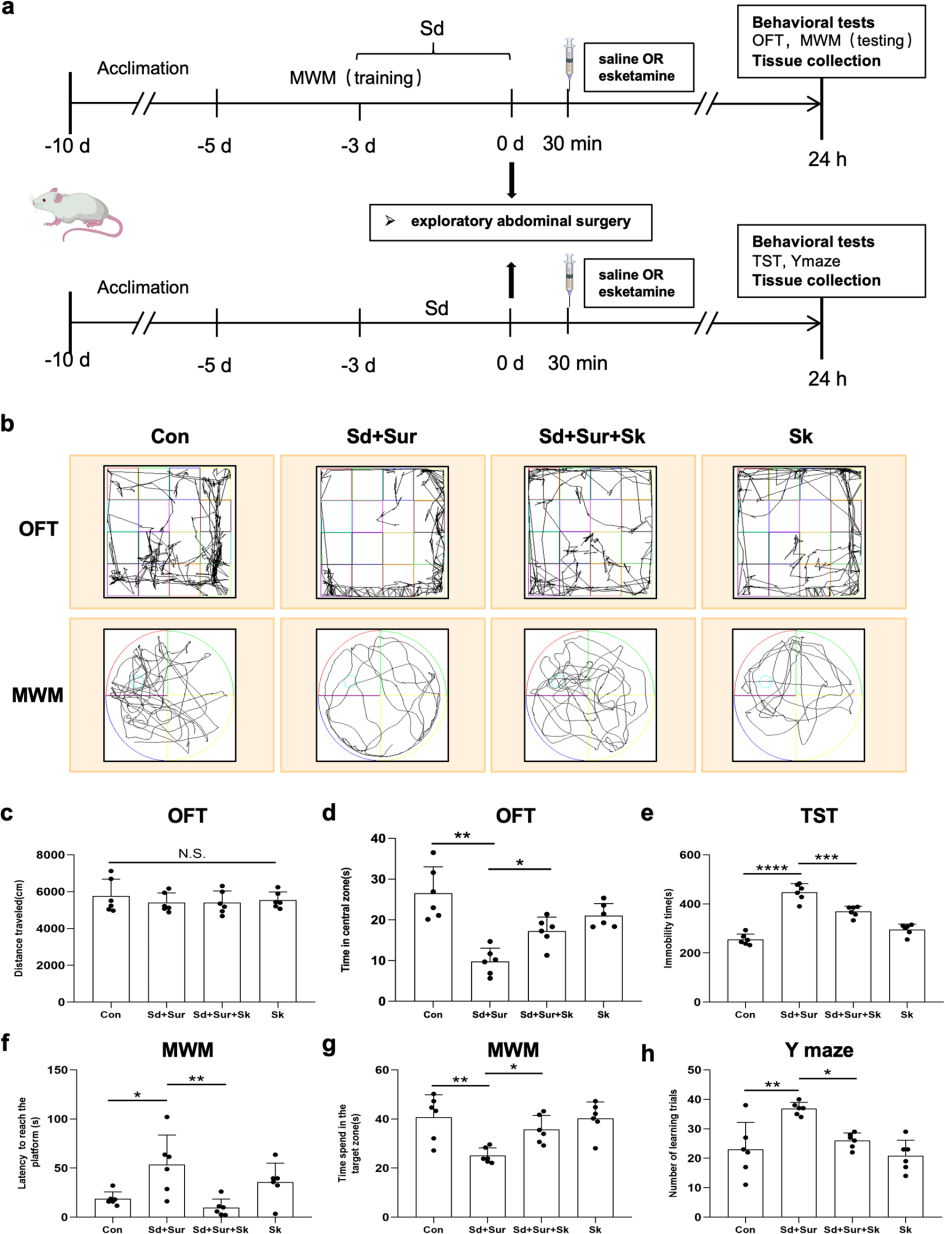
Esketamine relieved postoperative cognitive impairment and attenuated postoperative depression-like symptoms of Sd rats. **a** The schedule of experiment 2 procedure. **b** The track plot of the OFT and MWM performances of rats. OFT: **c** the overall travel distance and **d** time spent in the central zones. TST: **e** the immobility time. MWM: **f** latency; **g** time spent in the target zone. Y maze: **h** number of learning trials. Data are shown as mean ± S.E.M of six rats in each group. N.S: *p* > 0.05; *p < 0.05; **p < 0.01; ***p < 0.001; ****p < 0.0001

As described above, sleep disturbance aggravated POCD and POD. The effects of esketamine on postoperative symptoms were addressed in a sleep-disturbance rat model of exploratory laparotomy under anaesthesia. Behavioural experiments were performed 24 h after the surgery. The schematic timeline of the experiment is shown in Fig. 3a. The track plots for the OFT and MWM are presented in Fig. 3b. There were no significant differences in the total distance travelled in the OFT, demonstrating no difference in motor activities among the four groups of rats (Fig. 3c). In the OFT, esketamine treatment increased the time spent in the central zones compared with that of the control and sleep disturbance groups (Fig. 3d). In the TST, the immobility time of the Sd + Sur group was significantly increased compared with that of the control group, and esketamine treatment led to noticeable mitigation of the immobility time (Fig. 3e). The above results indicated that postoperative administration of esketamine could alleviate postoperative depression-like behaviours induced by sleep disturbance and surgery. MWM is primarily a test of spatial learning and memory. We found that sleep disturbance impaired spatial learning memory in rats because the latency was extended during the training phase of days 4–5 in the rat groups that receiving sleep disturbance (Fig. S1c). In the probe trails, there was no significant difference in swimming speed among the four groups (Fig. S1d). Meanwhile, esketamine treatment significantly shortened the escape latency compared to the Sd + Sur groups (Fig. 3f). In addition, the time spent in the target zone of Sd + Sur rats was overtly decreased compared with that of the control group, whereas esketamine treatment led to an increase (Fig. 3g). The Y maze was performed to test the learning ability of the rats in different groups. Compared with controls, rats in the Sd + Sur group had more learning trials, indicating impaired cognitive dysfunction. However, there was a decrease in learning trials after esketamine treatment (Fig. 3h). Taken together, postoperative esketamine treatment effectively alleviated postoperative cognitive impairment and attenuated depression-like symptoms aggravated by intermittent sleep disturbance in aged rats.

### Esketamine Relieved Sleep Disturbance-Augmented Microglial Activation and Inhibits the Activation of the NF-κB Pathway in the Hippocampus of Aged Rats

**Fig. 4:**
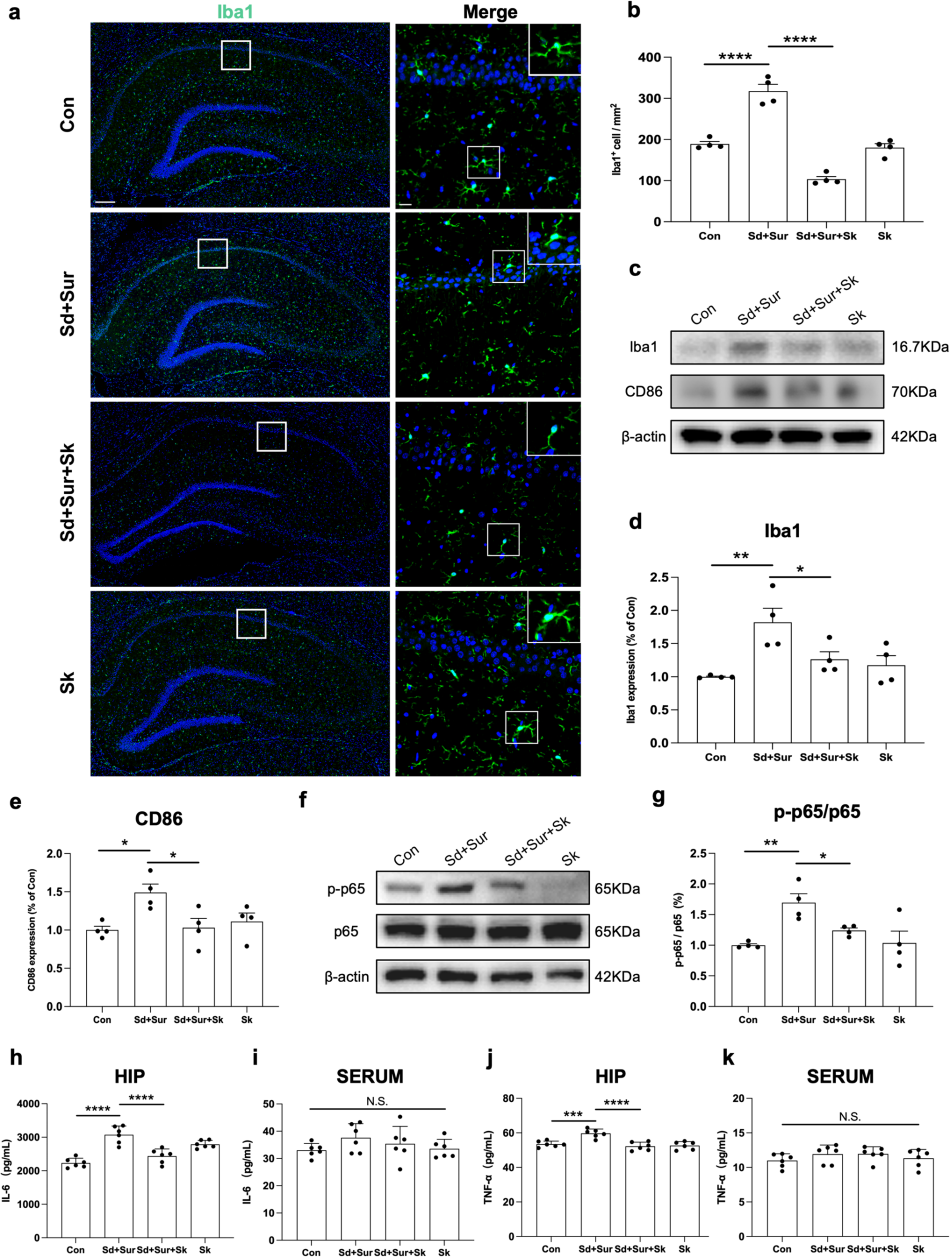
Esketamine inhibited microglial overactivation and M1-polarization. **a** Representative photomicrographs of Iba1 staining in the hippocampus of four groups. Scale bars 200 μm and 20 μm. **b** The number of Iba1-positive cells in hippocampus (*n* = 4 per group). **c**–**g** Representative results Iba1, CD86, and p-p65/p65 were obtained and quantification were expressed as the ratio (in percentage) of the control group. β-actin was used as loading control (*n* = 4 per group). **h** The levels of IL-6 in the hippocampus and **i** serum were detected by ELISA. **j** The levels of TNF-α in the hippocampus and **k** serum (*n* = 6 per group). All data were expressed as means ± SEM. N.S. *p* > 0.05; *p < 0.05; **p < 0.01; ***p < 0.001; ****p < 0.0001

Microglial activation correlates with increased depressive-like behaviours [34] as well as cognitive impairments [35]. As a result, we investigated the effects of esketamine on microglial activation by immunofluorescence for the microglia-specific marker Iba-1 (Fig. 4a). Our results indicated that preoperative sleep disturbance increased the number of microglia (Fig. 4b) and promoted the activation of microglia characterized by shortened and thickened processes and a more ameboid morphology. Meanwhile, rats that received anaesthesia and esketamine injections showed fewer Iba1-positive cells in hippocampal tissue and ramified processes with lower CD86 expression, as was confirmed by Western blot (Fig. 4c–e). Activation of the NF-κB pathway is closely related to the neuroinflammatory response and to the expression of proinflammatory cytokines. The immunocontent of p-p65 and p65 was evaluated in the hippocampus (Fig. 4 f and g). The results showed that preoperative sleep disturbance increased the expression of phosphorylated NF-κB, while esketamine treatment effectively alleviated phosphorylation. In addition, NF-κB-related inflammatory mediators were measured. We tested the levels of the proinflammatory cytokines IL-6 and TNF-α in the hippocampus and peripheral blood (Fig. 4h–k) 24 h after exploratory abdominal surgery by ELISA. We observed that rats in the Sd + Sur group presented a higher level of IL-6 and TNF-α in the hippocampus compared with the control group, and esketamine treatment effectively prevented the increased levels of the proinflammatory cytokines. In addition, esketamine treatment reduced the IL-6 and TNF-α levels in peripheral blood, although there was no statistical difference.

### Esketamine Prevents the Decreased BDNF-TrkB Signalling and Inhibits Inflammatory Responses Induced by Preoperative Sleep Disturbance and Surgery in the Hippocampus of Aged Rats

**Fig. 5:**
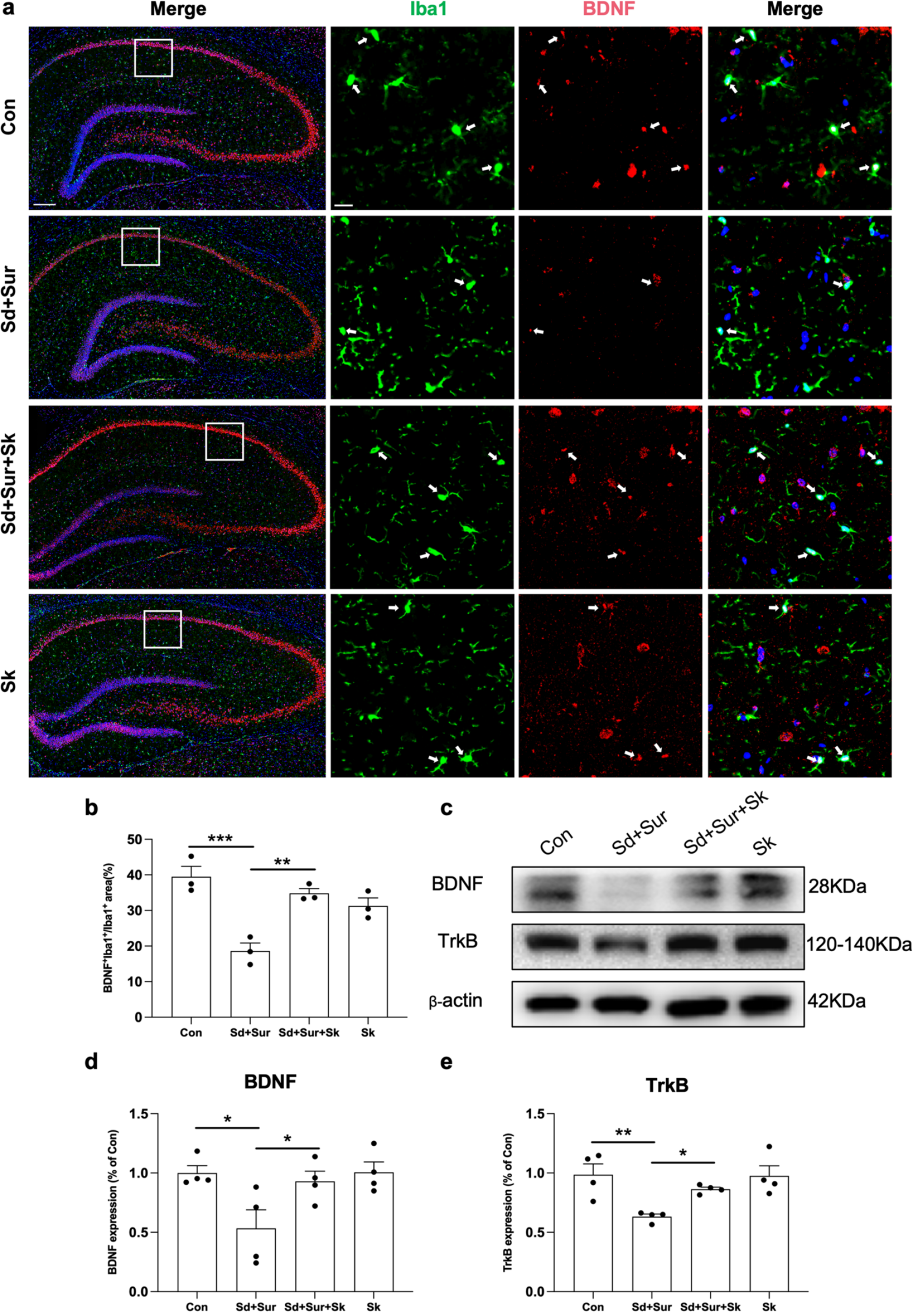
Esketamine restored BDNF-TrkB signalling disruption and inhibited neuroinflammatory responses after the sleep disruption and surgery. **a** Representative dual immunofluorescent images of Iba1/BDNF in the hippocampus of four groups. Scale bars 200 μm and 20 μm. **b** The percent areas of BDNF^+^ Iba1^+^/Iba1^+^ in the in right and left hippocampus in Con, Sur, and Sd + Sur groups (*n* = 3 per group). **c** Representative results were obtained from an experiment of BDNF and TrkB. **d**, **e** Quantification of BDNF and TrkB was expressed as the ratio (in percentage) of the control group (*n* = 4 per group). *p < 0.05; **p < 0.01; ***p < 0.001; ****p < 0.0001

Mounting evidence suggests that the decreased expression of the BDNF-TrkB signalling pathway is involved in the pathogenesis of POCD [36] and depression [37]. To further identify the effect of esketamine on the expression of BDNF, we performed double-labelling immunofluorescence for Iba1 and BDNF (Fig. 5a). Dual immunofluorescence staining showed that BDNF^+^ signals were profusely expressed in Iba1^+^ cells in the control group but less expressed in Iba1^+^ cells in the sleep disturbance before surgery group. The BDNF^+^ areas within Iba-1^+^ cells of the Sd + Sur group were much smaller than those of Con group (Fig. 5b). Moreover, esketamine treatment prevented the decrease in BDNF in Iba1^+^ cells in the hippocampus. Similarly, Western blotting showed the associated trends of BDNF (Fig. 5c, d). Furthermore, the immunocontent of TrkB was measured by Western blotting (Fig. 5c). Esketamine treatment effectively prevented the decrease in TrkB induced by preoperative sleep disturbance and surgery (Fig. 5e).

### Esketamine Prevents the Hippocampal Dendritic Spine Loss Induced by Sleep Disturbance Before Surgery

**Fig. 6:**
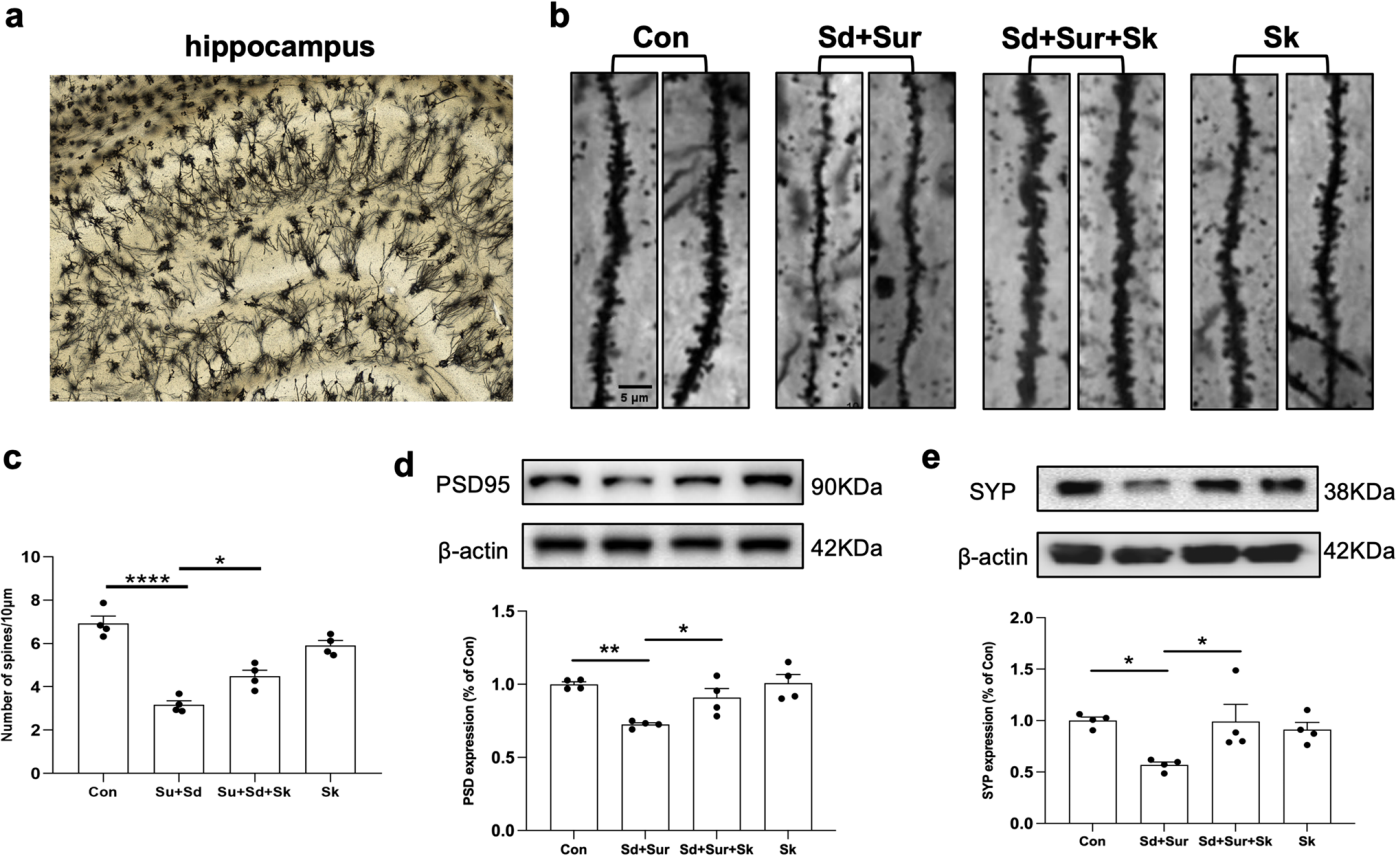
Esketamine prevented hippocampal synaptic plasticity damage after sleep disturbance and surgery. **a** A hippocampal profile image of Golgi-Cox staining. **b** Representative dendritic spine density of the hippocampus of the four groups. Scale bar 5 μm. **c** Quantitation of the dendritic spine density of the four groups (*n* = 4 per group). **d**, **e** Representative results obtained from an experiment of PSD95 and SYP and the quantification of PSD-95 and SYP were expressed as the ratio (in percentage) of the control group. β-actin was used as a loading control (*n* = 4 per group). All data were expressed as means ± SEM. *p < 0.05; **p < 0.01; ***p < 0.001; ****p < 0.0001

Dendritic spines, which are small extensions on dendrites, make up the postsynaptic component of excitatory synapses in the brain [38]. The morphology of the spines is an indicator of the stability, plasticity, and strength of associated synapses. The regulation of dendritic spine density contributes to depression and memory formation [39]. Therefore, to further explore the effect of esketamine on dendritic spines, we used Golgi-Cox staining to assess dendritic branching and spine density in neurons of the hippocampus. Our data indicated that the dendritic spine density was significantly reduced after sleep disturbance and surgery, and esketamine attenuated dendritic spine loss (Fig. 6c). Furthermore, sleep disturbance and surgical exposure significantly decreased the expression of PSD95 and SYP. The observed decreases in the levels of synaptic proteins were markedly reversed by esketamine (Fig. 6 d and e).

### Esketamine Inhibited LPS-Induced Microglial Activation and M1 Polarization in Primary Microglia

**Fig. 7:**
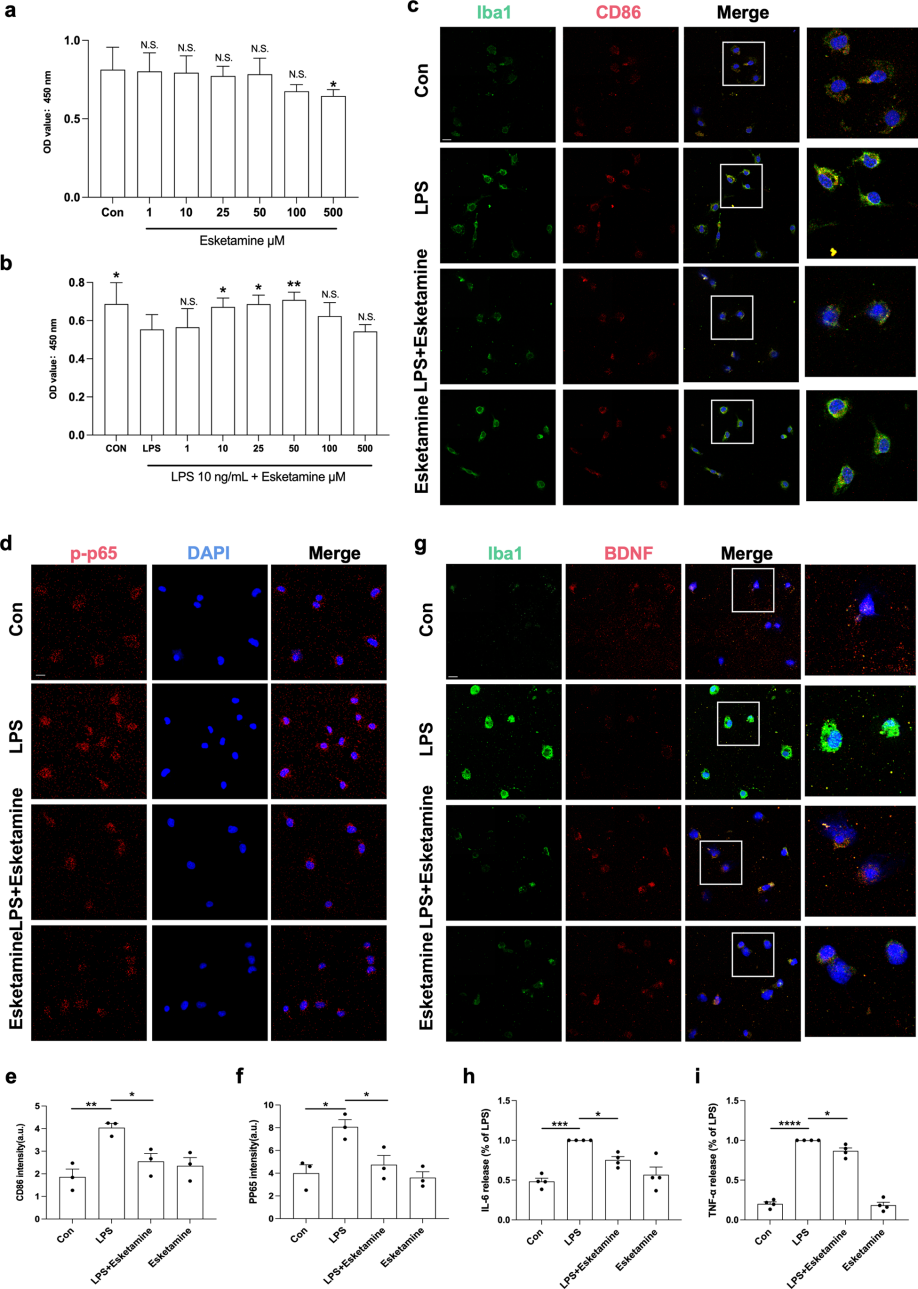
Esketamine alleviated LPS-induced microglial M1-polarization, increased of the immunocontent of inflammation markers, and prevented the decrease of BDNF of primary microglia. CCK8 assay was used to evaluate the survival cells after **a** esketamine treatment at 1, 10, 25, 50, 100, and 500μM for 24 h (* verse Con group) and **b** LPS (10 ng/mL) treatment for 30 min followed by the addition of esketamine (1-500μM) for 24 h (* verse LPS group). **c** Representative dual immunofluorescent images of Iba1/CD86 staining of primary microglia. Scale bar 20 μm. **d** Representative immunofluorescent images of p-p65 staining of primary microglia. Scale bar 20 μm. **e** Quantification of CD86 fluorescence (*n* = 3 per group). (**f**) Quantification of p-p65 fluorescence (n=3 per group). (**g**) Representative dual immunofluorescent images of Iba1/BDNF staining of primary microglia. Scale bar 20 μm. The levels of **h** IL-6 and **i** TNF-α of primary microglia were tested by ELISA (*n* = 4 per group). All data were expressed as means ± SEM. N.S.: *p* > 0.05; *p < 0.05; **p < 0.01; ***p < 0.001; ****p < 0.0001

To explore the proper concentration of esketamine stimulation, CCK-8 assays were used to detect the viability of primary microglial cells after treatment with various concentrations of esketamine for 24 h (Fig. 7a). Compared with the control group, esketamine at 1–100 μM showed no viability difference. Then, we treated microglia cells with LPS (10 ng/mL) for 30 min before adding various concentrations of esketamine for 24 h (Fig. 7b). Compared with the LPS group, the administration of 50 μM significantly improved cell viability. As a result, we chose 50 μM as our esketamine stimulation. To examine the effects of esketamine on the activation and polarization of microglia, we performed double-labelling immunofluorescence for Iba1 and CD86 (Fig. 7c). Esketamine administration significantly decreased the fluorescence intensity of Iba1 and M1 polarization induced by LPS in primary microglial cells (Fig. 7e).

### Esketamine Prevented Activation of the NF-κB Pathway and Microglia-Dependent BDNF Decrease Induced by LPS in Primary Microglia

To test the inhibitory effect of esketamine on the activation of the NF-κB pathway at the cellular level, we detected the expression of the p-p65 by immunostaining, and the results were in accordance with the above in vivo experiments. LPS treatment promoted the phosphorylation of p65, while the fluorescence intensity was significantly decreased after esketamine treatment (Fig. 7 d and f). Cell supernatant was collected, and the levels of the proinflammatory cytokines IL-6 and TNF-α were tested by ELISA (Fig. 7 h and i). We observed that esketamine treatment effectively prevented the increased levels of proinflammatory cytokines at the cellular level. To further confirm the effect of esketamine on the BDNF-TrkB pathways in primary microglia, we performed double-labelling immunofluorescence for Iba1 and BDNF. LPS-activated microglia significantly suppressed the expression of BDNF. These changes were significantly reversed by esketamine treatment (Fig. 7g).

## Discussion

In this study, we found that intermittent sleep deprivation could enhance postoperative complications both cognitively and emotionally of aged rats, and microglial M1 polarization was enhanced with lower levels of BDNF-TrkB signalling. Esketamine treatment significantly reversed the abnormalities in behavioural tests in the OFT, SPT, MWM, and Y maze by inhibiting the microglial M1 polarization and improving the levels of BDNF-TrkB signalling.

Sleep of normal quality and duration is essential for emotional brain processing and cognitive function [40]. Loss or restriction of sleep is associated with multiple detrimental consequences, especially in the elderly [41]. However, few studies have paid attention to sleep deprivation within the perioperative field. In our study, we employed an intermittent sleep deprivation rat model, as previously described, to simulate patients’ preoperative sleep status. The results showed that sleep disturbance markedly performed a pro-depressant effect and impaired the cognitive functions of aged rats, as shown in the MWM and Y maze. Ketamine (or RS-ketamine) is a racemic mixture containing equal parts of R-ketamine and S-ketamine. Emerging evidence has shown that the use of small doses of ketamine during surgery not only enhances the pharmacological efficacy of anaesthetics but also benefits postoperative cerebral complications [2]. S-ketamine has an approximately four-fold greater affinity for the NMDA receptor than R-ketamine [42]. In our study, the behavioural abnormalities were reversed after esketamine treatment. However, the underlying mechanisms have not been investigated.

The activation of the inflammatory response in the brain is a critical step in the pathogenesis of postoperative complications [35, 43]. The upregulation of inflammatory factors and activation of microglia lead to impairment of synaptic plasticity and neurogenesis, which are key aspects of pathophysiology of postoperative complications [7]. The hippocampus plays an important role in depression and cognitive function due to its high plasticity and stress sensitivity [44]. Hippocampal-dependent learning and memory are particularly vulnerable to surgery in aged animals [45, 46]. So, we focused on the hippocampus in rats. It is postulated that microglia are activated initially and become a major cellular source of a variety of proinflammatory cytokines in a rat model of neuroinflammation. Microglial M1-polarization causes the release of proinflammatory cytokines, such as TNF-α, IL-1β, and IL-6, to respond to stimuli [47]. Uncontrolled and prolonged M1-activated state contributes to additional neuronal damage [48]. Reducing the expression of proinflammatory factors through gene regulation or drug intervention can significantly improve postoperative abnormalities [49]. Our data showed that the level of Iba1 and phosphorylated p65 were significantly increased in the hippocampus of aged rats after sleep disturbance and surgery. Interestingly, esketamine prevented the activation of NF-κB pathway and microglial M1 polarization both in vivo and in vitro.

Brain-derived neurotrophic factor (BDNF) is among the most important neurotrophic factors released from nerve cells and microglia [50]. BDNF performs its neuroprotective functions mainly through binding to its high-affinity receptor tyrosine kinase B (TrkB) [51], while binding to the low-affinity p75 neurotrophin receptor (p75NTR) could activate the apoptosis cascade [52]. However, NF-κB plays an important role in BDNF-induced neuroprotection. Specifically, activated NF-κB can translocate into the nucleus, where it binds to the promoters on transcripts of the BDNF gene to influence BDNF transcription in microglia [47, 53, 54]. Moreover, some evidence proved that activated cAMP response element-binding protein (CREB) could inhibit NF-κB activity, which may affect the level of the binding of BDNF and TrkB [55, 56]. As shown in previous studies, lower levels of BDNF-TrkB signalling may contribute to NF-κB signalling activation[57, 58]. However, the relationship between BDNF and the NF-κB pathway has not been determined directly in POCD and POD. In our study, we found that esketamine could not only mediate the BDNF-TrkB pathway but also decrease the activation of the NF-κB pathway. Therefore, we speculate that BDNF can play a role in the postoperative brain protective effect of esketamine by inhibiting inflammatory pathways. The ANA-12 (TrkB antagonist) used in other studies was proven to inhibit the anti-inflammatory effect of esketamine [59]. Moreover, in animal models, changes in BDNF-TrkB signalling lead to a decrease in dendritic spines and arborisation and could ultimately contribute to neurotic dystrophy and degeneration [60–62]. In the current study, we found that esketamine could prevent synaptic dysfunction and the loss of synapse-associated proteins PSD95 and SYP, which have a close relationship with severe memory impairment and depressive behaviour [63].

There are some limitations in this study. Firstly, we mainly observed relatively short-term cognitive and emotional performance, but long-term effects remain unknown. Secondly, other brain regions, including prefrontal cortex, amygdala and hypothalamus, lateral habenula, and nucleus accumbens, have been associated with postoperative adverse events [59], which still needs to be explored in the future. In addition, the study was focused on changes of microglia post-surgery, neurons and other type of glial cells should be further investigated.

**Fig. 8:**
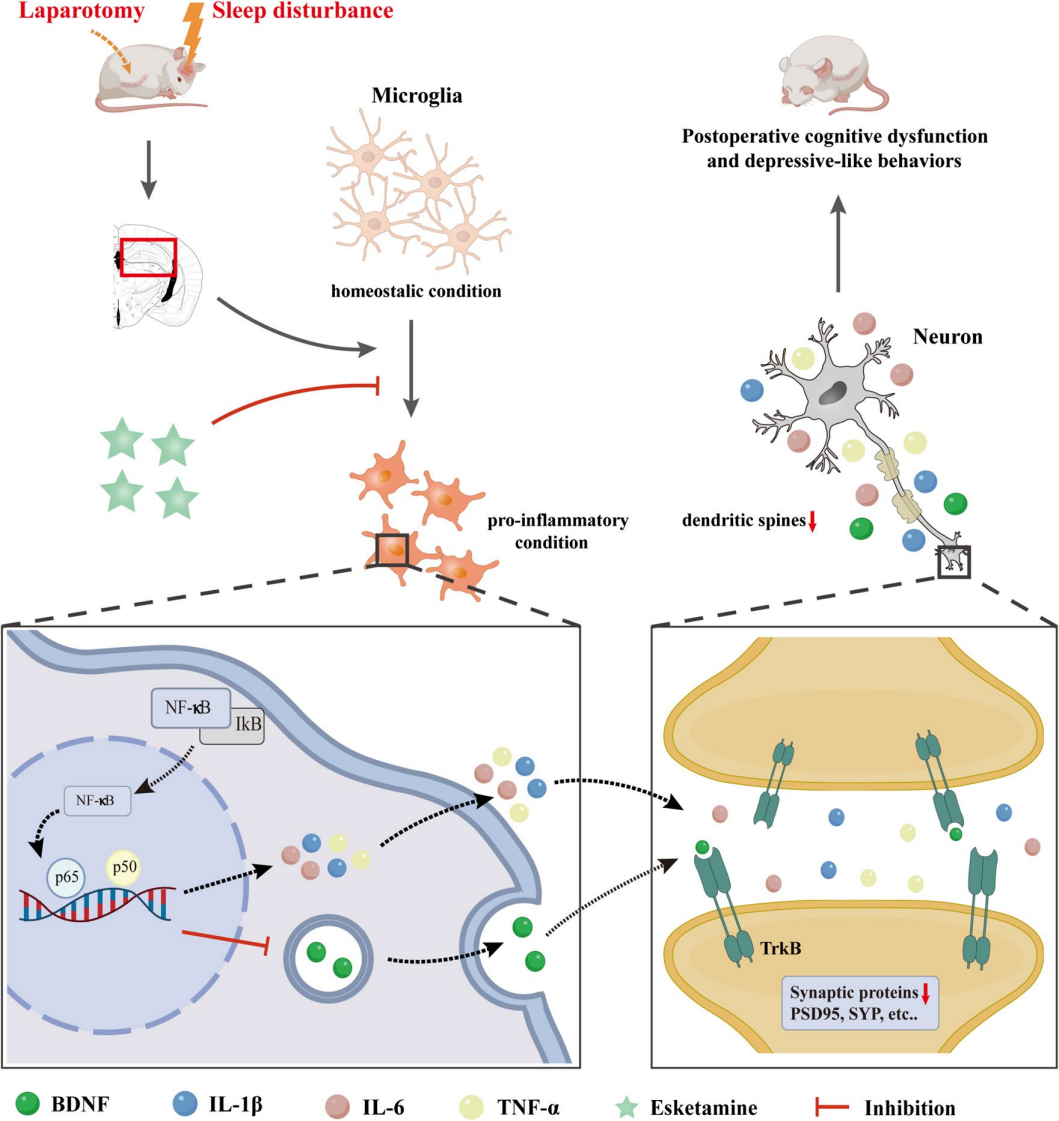
Schematic illustration of the mechanism underlying esketamine improves postoperative cognitive dysfunction and depressive-like symptoms

In summary, this study suggested that intermittent sleep disturbance before surgery exacerbated microglial activation, neuroinflammation, and microglial BDNF-TrkB signalling dysfunction induced by surgery, resulting in postoperative emotional changes and cognitive impairments of aged rats. Esketamine inhibited the M1 polarization of microglia and the inflammatory response and thus improved BDNF-TrkB signalling and impaired hippocampal synaptic plasticity (Fig. 8). These findings may provide novel ideas for the implication of preoperative preparations and the prevention of postoperative brain-related complications.

### Supplementary information

ESM 1 (DOCX 4587 kb)

## Data Availability

The datasets used and/or analysed during the current study are available from the corresponding author upon reasonable request.

## Abbreviations

CNS: Central nervous system
POD: Postoperative depression
POCD: Postoperative cognitive dysfunction
Sd: Preoperative sleep disturbance
Sk: Esketamine
Sur: Surgery
BSA: Bovine serum albumin
PBS: Phosphate-buffered saline
DAPI: 4′,6-diamidino-2-phenylindole
i.p.: Intraperitoneal
TST: Tail suspension test
OFT: Open field test
MWM: Morris water maze
WB: Western blotting
ELISA: Enzyme-linked immunosorbent assay
TNF-α: Tumour necrosis factor α
IL-1β: Interleukin 1 beta
IL-6: Interleukin-6
Iba-1: Ionized calcium-binding adaptor molecule-1
LPS: Lipopolysaccharides
BDNF: Brain-derived neurotrophic factor
TrkB: Tyrosine kinase receptor B
PSD95: Postsynaptic density protein 95
SYP: Synaptophysin
HIP: Hippocampus
OD: Optical density

## References

[1] Ghoneim MM, O’Hara MW (2016). Depression and postoperative complications: an overview. BMC Surg.

[2] Li S, Luo X, Hua D, Wang Y, Zhan G, Huang N (2019). Ketamine alleviates postoperative depression-like symptoms in susceptible mice: the role of BDNF-TrkB signaling. Front Pharmacol.

[3] Nickinson RS, Board TN, Kay PR (2009). Post-operative anxiety and depression levels in orthopaedic surgery: a study of 56 patients undergoing hip or knee arthroplasty. J Eval Clin Pract.

[4] Jiang M, Wang MH, Wang XB, Liu L, Wu JL, Yang XL (2016). Effect of intraoperative application of ketamine on postoperative depressed mood in patients undergoing elective orthopedic surgery. J Anesth.

[5] Muller A, Hase C, Pommnitz M, de Zwaan M (2019). Depression and suicide after bariatric surgery. Curr Psychiatry Rep.

[6] Uher R, Payne JL, Pavlova B, Perlis RH (2014). Major depressive disorder in Dsm-5: implications for clinical practice and research of changes from Dsm-Iv. Depress Anxiety.

[7] Kettenmann H, Hanisch UK, Noda M, Verkhratsky A (2011). Physiology of microglia. Physiol Rev.

[8] Enache D, Pariante CM, Mondelli V (2019). Markers of central inflammation in major depressive disorder: A systematic review and meta-analysis of studies examining cerebrospinal fluid, positron emission tomography and post-mortem brain tissue. Brain Behav Immun.

[9] Dowlati Y, Herrmann N, Swardfager W, Liu H, Sham L, Reim EK (2010). A meta-analysis of cytokines in major depression. Biol Psychiatry.

[10] Yang Y, Zhang M, Kang X, Jiang C, Zhang H, Wang P (2015). Thrombin-induced microglial activation impairs hippocampal neurogenesis and spatial memory ability in mice. Behav Brain Funct.

[11] Yirmiya R, Rimmerman N, Reshef R (2015). Depression as a microglial disease. Trends Neurosci.

[12] Takamura R, Watamura N, Nikkuni M, Ohshima T (2017). All-trans retinoic acid improved impaired proliferation of neural stem cells and suppressed microglial activation in the hippocampus in an Alzheimer’s mouse model. J Neurosci Res.

[13] Prieto GA, Cotman CW (2017). Cytokines and cytokine networks target neurons to modulate long-term potentiation. Cytokine Growth Factor Rev.

[14] Ghosh A, Carnahan J, Greenberg ME (1994). Requirement for BDNF in activity-dependent survival of cortical neurons. Science.

[15] Sun X, Zeng H, Wang Q, Yu Q, Wu J, Feng Y (2018). Glycyrrhizin ameliorates inflammatory pain by inhibiting microglial activation-mediated inflammatory response via blockage of the HMGB1-TLR4-NF-kB pathway. Exp Cell Res.

[16] Muller J, Pentyala S, Dilger J, Pentyala S (2016). Ketamine enantiomers in the rapid and sustained antidepressant effects. Ther Adv Psychopharmacol.

[17] Eberl S, Koers L, van Hooft J, de Jong E, Hermanides J, Hollmann MW (2020). The effectiveness of a low-dose esketamine versus an alfentanil adjunct to propofol sedation during endoscopic retrograde cholangiopancreatography A randomised controlled multicentre trial. Eur J Anaesthesiol.

[18] Tan SJ, Wang Y, Chen K, Long ZF, Zou J (2017). Ketamine alleviates depressive-like behaviors via down-regulating inflammatory cytokines induced by chronic restraint stress in mice. Biol Pharm Bull.

[19] Hashimoto K (2019). Rapid-acting antidepressant ketamine, its metabolites and other candidates: a historical overview and future perspective. Psychiatry Clin Neurosci.

[20] Toth LA, Bhargava P (2013). Animal models of sleep disorders. Comp Med.

[21] Ahmadi-Soleimani SM, Azizi H, Abbasi-Mazar A (2021). Intermittent REM sleep deprivation attenuates the development of morphine tolerance and dependence in male rats. Neurosci Lett.

[22] Naidoo N, Ferber M, Master M, Zhu Y, Pack AI (2008). Aging impairs the unfolded protein response to sleep deprivation and leads to proapoptotic signaling. J Neurosci.

[23] Masaki Y, Kashiwagi Y, Watabe H, Abe K (2019). (R)- and (S)-ketamine induce differential fMRI responses in conscious rats. Synapse.

[24] Yang C, Shirayama Y, Zhang JC, Ren Q, Yao W, Ma M (2015). R-ketamine: a rapid-onset and sustained antidepressant without psychotomimetic side effects. Transl Psychiatry.

[25] Fukumoto K, Toki H, Iijima M, Hashihayata T, Yamaguchi JI, Hashimoto K (2017). Antidepressant potential of (R)-ketamine in rodent models: comparison with (S)-ketamine. J Pharmacol Exp Ther.

[26] Gordon R, Hogan CE, Neal ML, Anantharam V, Kanthasamy AG, Kanthasamy A (2011). A simple magnetic separation method for high-yield isolation of pure primary microglia. J Neurosci Methods.

[27] Zhang K, Yang C, Chang L, Sakamoto A, Suzuki T, Fujita Y (2020). Essential role of microglial transforming growth factor-beta1 in antidepressant actions of (R)-ketamine and the novel antidepressant TGF-beta1. Transl Psychiatry.

[28] Sofroniew MV (2015). Astrocyte barriers to neurotoxic inflammation. Nat Rev Neurosci.

[29] Xie X, Shen Z, Hu C, Zhang K, Guo M, Wang F (2021). Dexmedetomidine ameliorates postoperative cognitive dysfunction in aged mice. Neurochem Res.

[30] He J, Liu T, Li Y, Mi X, Han D, Yang N (2021). JNK inhibition alleviates delayed neurocognitive recovery after surgery by limiting microglia pyroptosis. Int Immunopharmacol.

[31] Yang C, Kobayashi S, Nakao K, Dong C, Han M, Qu Y (2018). AMPA receptor activation-independent antidepressant actions of ketamine metabolite (S)-norketamine. Biol Psychiatry.

[32] Vorhees CV, Williams MT (2006). Morris water maze: procedures for assessing spatial and related forms of learning and memory. Nat Protoc.

[33] De Luca C, Colangelo AM, Alberghina L, Papa M (2018). Neuro-immune hemostasis: homeostasis and diseases in the central nervous system. Front Cell Neurosci.

[34] Kreisel T, Frank MG, Licht T, Reshef R, Ben-Menachem-Zidon O, Baratta MV (2014). Dynamic microglial alterations underlie stress-induced depressive-like behavior and suppressed neurogenesis. Mol Psychiatry.

[35] Alam A, Hana Z, Jin Z, Suen KC, Ma D (2018). Surgery, neuroinflammation and cognitive impairment. EBioMedicine.

[36] Qiu LL, Ji MH, Zhang H, Yang JJ, Sun XR, Tang H (2016). NADPH oxidase 2-derived reactive oxygen species in the hippocampus might contribute to microglial activation in postoperative cognitive dysfunction in aged mice. Brain Behav Immun.

[37] Shirayama Y, Chen AC, Nakagawa S, Russell DS, Duman RS (2002) Brain-derived neurotrophic factor produces antidepressant effects in behavioral models of depression. J Neurosci 22(8): 3251-3261 10.1523/JNEUROSCI.22-08-03251.2002 PMC6757539 11943826

[38] Runge K, Cardoso C, de Chevigny A (2020). Dendritic spine plasticity: function and mechanisms. Front Synaptic Neurosci.

[39] Yang M, Luo CH, Zhu YQ, Liu YC, An YJ, Iqbal J (2020). 7,8-Dihydroxy-4-methylcoumarin reverses depression model-induced depression-like behaviors and alteration of dendritic spines in the mood circuits. Psychoneuroendocrinology.

[40] Hou JB, Shen QN, Wan X, Zhao B, Wu Y, Xia ZY (2019). REM sleep deprivation-induced circadian clock gene abnormalities participate in hippocampal-dependent memory impairment by enhancing inflammation in rats undergoing sevoflurane inhalation. Behav Brain Res.

[41] Musiek ES, Holtzman DM (2016). Mechanisms linking circadian clocks, sleep, and neurodegeneration. Science.

[42] Domino EF (2010). Taming the ketamine tiger. Anesthesiology.

[43] Meyer JH, Cervenka S, Kim MJ, Kreisl WC, Henter ID, Innis RB (2020). Neuroinflammation in psychiatric disorders: PET imaging and promising new targets. Lancet Psychiatry.

[44] Masi G, Brovedani P (2011). The hippocampus, neurotrophic factors and depression: possible implications for the pharmacotherapy of depression. CNS Drugs.

[45] Dellarole A, Morton P, Brambilla R, Walters W, Summers S, Bernardes D (2014). Neuropathic pain-induced depressive-like behavior and hippocampal neurogenesis and plasticity are dependent on TNFR1 signaling. Brain Behav Immun.

[46] Micheli L, Ceccarelli M, D’Andrea G, Tirone F (2018). Depression and adult neurogenesis: positive effects of the antidepressant fluoxetine and of physical exercise. Brain Res Bull.

[47] Patterson SL (2015). Immune dysregulation and cognitive vulnerability in the aging brain: interactions of microglia, IL-1beta, BDNF and synaptic plasticity. Neuropharmacology.

[48] Martinez FO, Gordon S (2014). The M1 and M2 paradigm of macrophage activation: time for reassessment. F1000Prime Rep.

[49] Xu X, Xiao X, Yan Y, Zhang T (2021). Activation of liver X receptors prevents emotional and cognitive dysfunction by suppressing microglial M1-polarization and restoring synaptic plasticity in the hippocampus of mice. Brain Behav Immun.

[50] Tyler WJ, Alonso M, Bramham CR, Pozzo-Miller LD (2002). From acquisition to consolidation: on the role of brain-derived neurotrophic factor signaling in hippocampal-dependent learning. Learn Mem.

[51] Teixeira AL, Barbosa IG, Diniz BS, Kummer A (2010). Circulating levels of brain-derived neurotrophic factor: correlation with mood, cognition and motor function. Biomark Med.

[52] Barker PA (2009). Whither proBDNF?. Nat Neurosci.

[53] Aid T, Kazantseva A, Piirsoo M, Palm K, Timmusk T (2007). Mouse and rat BDNF gene structure and expression revisited. J Neurosci Res.

[54] Gao L, Zhang Y, Sterling K, Song W (2022). Brain-derived neurotrophic factor in Alzheimer’s disease and its pharmaceutical potential. Transl Neurodegener.

[55] Wen AY, Sakamoto KM, Miller LS (2010). The role of the transcription factor CREB in immune function. J Immunol.

[56] Wu SY, Pan BS, Tsai SF, Chiang YT, Huang BM, Mo FE (2020). BDNF reverses aging-related microglial activation. J Neuroinflammation.

[57] Bi QR, Hou JJ, Qi P, Ma CH, Shen Y, Feng RH (2016). Venenum bufonis induces rat neuroinflammation by activiating NF-kappaB pathway and attenuation of BDNF. J Ethnopharmacol.

[58] Kunugi H, Hori H, Adachi N, Numakawa T (2010). Interface between hypothalamic-pituitary-adrenal axis and brain-derived neurotrophic factor in depression. Psychiatry Clin Neurosci.

[59] Wang TY, Weng HD, Zhou HJ, Yang ZC, Tian ZY, Xi BA (2022). Esketamine alleviates postoperative depression-like behavior through anti-inflammatory actions in mouse prefrontal cortex. J Affect Disord.

[60] Linnarsson S, Bjorklund A, Ernfors P (1997). Learning deficit in BDNF mutant mice. Eur J Neurosci.

[61] Lyons WE, Mamounas LA, Ricaurte GA, Coppola V, Reid SW, Bora SH (1999). Brain-derived neurotrophic factor-deficient mice develop aggressiveness and hyperphagia in conjunction with brain serotonergic abnormalities. Proc Natl Acad Sci USA.

[62] Ferrer I, Marin C, Rey MJ, Ribalta T, Goutan E, Blanco R (1999). BDNF and full-length and truncated TrkB expression in Alzheimer disease. Implications in therapeutic strategies. J Neuropathol Exp Neurol.

[63] Taliaz D, Stall N, Dar DE, Zangen A (2010). Knockdown of brain-derived neurotrophic factor in specific brain sites precipitates behaviors associated with depression and reduces neurogenesis. Mol Psychiatry.

