# Supplementary material for: Esketamine Prevents Postoperative Emotional and Cognitive Dysfunction by Suppressing Microglial M1 Polarization and Regulating the BDNF-TrkB Pathway in Ageing Rats with Preoperative Sleep Disturbance
Source: Mol Neurobiol. 2024 Jan 15;61(8):5680–98. doi: 10.1007/s12035-023-03860-4 (PMC11249437; doi:10.1007/s12035-023-03860-4)
Supplement: Supplementary file 1 — (DOCX 4587 kb) [file 12035_2023_3860_MOESM1_ESM.docx]

Supplementary materials 1


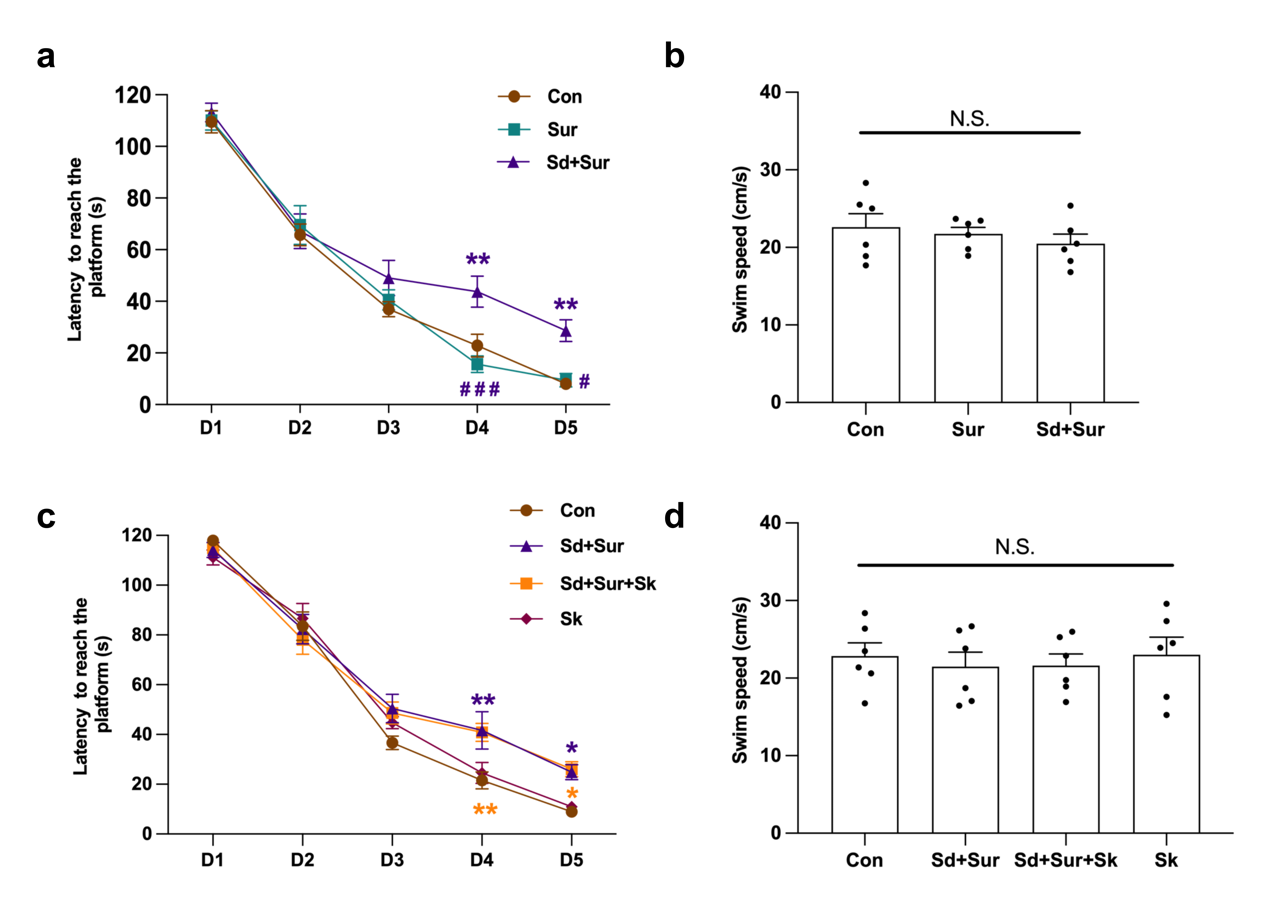


**Sleep disturbance enhanced postoperative cognitive impairment and postoperative depression-like symptoms.** (**a**) Escape latency during the training phase. **P < 0.01 vs. Con, ^#^P < 0.05, ^###^P < 0.001 vs. Sur; (**b**) average speed during the testing phase. N.S: p>0.05. Data are shown as mean ± S.E.M of 6 rats in each group.

**Esketamine relieved postoperative cognitive impairment and attenuated postoperative depression-like symptoms of Sd rats.** (**c**) Escape latency during the training phase. *P < 0.05, **P < 0.01 vs. Con; (**d**) average speed during the testing phase. N.S: p>0.05. Data are shown as mean ± S.E.M of 6 rats in each group.
